# Employing cognitive interviewing to evaluate, improve and validate items for measuring the health-related quality of life of women diagnosed with ovarian cancer

**DOI:** 10.1186/s12905-022-01966-w

**Published:** 2022-09-27

**Authors:** Sharolin Ann Boban, Caroline Bulsara, Jim Codde, Paul A. Cohen, Jenny Downs

**Affiliations:** 1 School of Health Sciences, University of Notre Dame Australia, Fremantle, WA Australia; 2 Institute for Health Research, University of Notre Dame Australia, Fremantle, WA Australia; 3 Division of Obstetrics and Gynecology, Faculty of Health and Medical Sciences, University of Western Australia, Perth, WA Australia; 4 Telethon Kids Institute, University of Western Australia, Nedlands, WA Australia

**Keywords:** Cognitive interviewing, Health-related quality of life, Ovarian cancer, Patient reported outcome measures

## Abstract

**Background:**

Use of patient-reported outcome measures in clinical settings facilitate the delivery of better health care to improve patient health outcomes. Previously collected qualitative data indicated themes that could inform items for a health-related quality of life measure. This study investigated the content validity of items for inclusion in a new health-related quality of life measure suitable for patients with ovarian cancer.

**Methods:**

Cognitive interviewing techniques were used with fourteen women diagnosed with ovarian cancer and at different times since diagnosis, to evaluate items derived from the previously collected qualitative dataset. A set of draft items was administered via telephone, Zoom and WhatsApp app together with questions on item meaning and wording. Interviews were transcribed and thematically analysed.

**Results:**

Four broad themes emerged in relation to the questionnaire construction and comprehension of items: intent and clarity, wording, relevance and context, and overall questionnaire construct. All draft items were adjusted based on the interview findings. A final set of 38 health-related quality of life items comprised 7 items describing physical health and functioning, 21 describing emotional wellbeing and 10 items describing social wellbeing; each rated on a five-point frequency response scale.

**Conclusion:**

The items reflected a range of personal experiences associated with the patient clinical journey, creating a health-related quality of life tool specific to women diagnosed with ovarian cancer. The cognitive interviewing process established content validity for the tool, thereby, preparing it for field testing and evaluation of its psychometric properties. This study highlighted the fundamental role of cognitive interviewing during health-related quality of life questionnaire development to ensure that item content is grounded in patient feelings, functioning and meaning.

**Supplementary Information:**

The online version contains supplementary material available at 10.1186/s12905-022-01966-w.

## Background

The goal of this study was to examine the meaning and coherence of items which formed a health-related quality of life questionnaire for patients with ovarian cancer. Ovarian cancer (OC) is the most lethal gynaecological malignancy. Globally, 230 000 women are diagnosed with OC and 150 000 die of the disease each year [1]. In Australia, OC is the eighth most commonly diagnosed cancer among women with approximately 1500 new cases diagnosed every year and a five-year relative survival of only 46% [2]. OC survivors remain at high risk of relapse and fear of cancer recurrence which may lead to significant anxiety and psychological morbidity [3]. Many OC survivors report moderate to severe symptoms such as peripheral neuropathy and fatigue two years after completing treatment, and may experience a disease recurrence, or developing a new primary cancer, all of which can influence health-related quality of life (HRQOL) [4, 5].

Patient reported outcomes are defined as “any report of the status of a patient’s health condition that comes directly from the patient, without interpretation of the patient’s response by a clinician or anyone else” [6, 7]. Patient reported outcomes are collected using tools and/or instruments called patient reported outcome measures (PROMs) [8]. Incorporating PROMs into clinical settings is believed to enhance the delivery of health care [9] and achievement of patient health outcomes [10].

Clinical trials involving cancer patients often utilize general PROMs, (for example, the Short Form Survey 36 (SF-36)) and validated tools for specific cancer types, including gynaecologic cancers [11]. Whilst commonly used in research, PROMs are less often used in routine clinical practice [9], and important information on functional recovery and HRQOL could be missed.

The European Organization for Research and Treatment of Cancer Quality of Life Questionnaire for Cancer Patients (EORTC QLQ-C30) and Functional Assessment of Chronic Illness Therapy Functional Assessment of Cancer Therapy-General (FACIT FACT-G) are the two most widely used cancer-specific HRQOL questionnaires and can be augmented by site- and/or treatment-specific modules [11]. Both EORTC and FACIT have OC-specific modules including EORTC QLQ-OV28, FACT-O and FACT-ovarian cancer symptom index (FOSI) [12]. The EORTC QLQ-C30 questionnaire contains 30 items which assess functioning, global HRQOL, and cancer-related symptoms. It is complemented by a OC module, QLQ-OV28, which contains a further 28 items including body image, abdominal/gastrointestinal symptoms, hormonal/menopausal symptoms [13]. The FACT-O questionnaire comprises 27 items from FACIT FACT-G to cover four core domains of wellbeing with an additional 12 items specific to OC. The FOSI is a shorter, more focused subset of the FACT-O items that includes three subscales: disease/treatment-related symptoms and general function/wellbeing [14]. More recently, King et al. developed a tool, the Measure of Ovarian Symptoms and Treatment concerns (MOST), to assess patient-reported symptom burden as an end point in clinical trials. This tool consists of 24 items including abdominal/disease/treatment-related symptoms, psychological symptoms and MOST-Well-being [15].

Whilst each of these tools seek to describe experiences of women with OC, each were developed using different methodological approaches and this could explain variations in their content [16]. For example, the FACT-O was developed to assess symptoms and quality of life and semi-structured interviews were conducted with five health professionals (nurses) and 17 OC patients with varying disease severity. Items were then reviewed by a panel of experts [17]. Additional to symptoms, the items reflect psychosocial aspects such as “appearance of my body,” “able to feel like a woman” [17]. Similarly, to the FACT-O, the QLQ-OV28 focused on measuring symptoms, both general and disease specific symptoms. Unlike the MOST which primarily focused on symptom benefit in women with symptomatic OC, neither QLQ-OV28 nor the FACT-O were specifically developed and validated in patients with platinum resistant recurrent OC, where the aim of treatment is symptom benefit and palliation. Similarly, differences exist in the level and stage of patient involvement in the development of PROMs [13, 14]. The importance of this is seen in the work of Kirwan [18, 19] and illustrated by Friedlander who showed clear differences between the level of importance of symptoms reported by symptom benefit in women with symptomatic OC.

We previously conducted semi-structed interviews and focus groups with women with OC and identified key experiences and priorities for women diagnosed with OC. Findings included challenges related to diagnosis and treatment, adjustments in their relationships with family and/or friends, financial issues, some difficulties in their relationships with health professionals and comment on useful coping strategies. Through the use of template thematic analysis, these findings were further developed into a set of items that could be useful for the development of a values-based OC PROM (Additional file 1) [20].

Establishing content validity is a fundamental first step in determining whether an outcome measure is fit for purpose [21]. Using cognitive interviewing (CI), the purpose of this study was to refine, and content validate the items/statements derived from the qualitative data collected in our previous study. We also aimed to examine whether additional items from the dataset could contribute to a broader questionnaire on factors related to HRQOL (Additional file 2).

## Methods

This study employed cognitive interviewing (CI) with the integration of concurrent think-aloud (CTA) procedure. CI is a method whereby items and contents and response processes can be assessed and validated [22], ensuring the content clarity and relevance of the items and response categories [23, 24]. The CTA procedure is a research method in which respondents speak aloud about their thoughts as they complete each questionnaire item in regard to personal understanding of the items [25, 26]. Thus, this interview procedure was implemented to identify items where respondent interpretation and the developer’s intentions were dissimilar and to identify ways in which those items can be modified based on the responses given [23, 25]. The questionnaire and interview guide used in the study was developed for this study and is provided as Additional file 2 and in Table 1. This study was granted ethics approval by the Human Research Ethics Committee at University of Notre Dame Australia (UNDA) (2020-010F) and our study complied with the Declaration of Helsinki.

**Table 1:** Probing questions used during the CTA procedure

Context	Question format	Example
HOW	How did you respond to that statement?	*“How would you rate that statement?”*
WHY	Why did you respond in that way?	*“Why did you agree but not strongly agree?”*
REWORD	Is there any other way to reword the statement?	*“You were confused with that statement. So, would you reword that statement?”*

### Recruitment procedures and study population

Our previous study incorporated input and guidance from relevant community support organisations, primarily Ovarian Cancer Australia (OCA) and Cancer Council Western Australia (CCWA). This study also worked in partnership with CCWA, OCA and the Australia New Zealand Gynaecological Oncology Group-Survivors Teaching Students, in recruiting participants through advertisements distributed through the media and relevant agencies. The recruitment process was slow as it coincided with the COVID-19 pandemic. Additional participants were recruited through King Edward Memorial Hospital and Solaris Cancer Care, a patient support organisation, in Perth, Western Australia. All participants who expressed interest in participating in the study were contacted directly by the UNDA researcher (SB) to schedule an interview at a mutually convenient time.

Purposive sampling technique which utilized a maximum variation strategy, aiming to represent variation in the stage of OC, treatment received, demographic and socioeconomic characteristics was employed. This sampling technique provides insights and in-depth knowledge regarding individuals’ experience in different circumstances [27]. Participants were women diagnosed with OC, older than 18 years and were proficient in English.

### Data collection

Participants were provided with all relevant documents including a consent form, participant information sheet and interview schedule (provided as Additional file 3), and signed consent was received prior to interview commencement. Due to the COVID-19 pandemic, interviews were conducted using telephone or video (Zoom/Skype/WhatsApp App) according to the participant’s preference. Identified statements from our previous qualitative study were tested by conducting CI using the CTA procedure (Table 1). The focus of applying CTA was not limited to determining certain words or response categories of the items but also to identify how and why respondents answered each item. Prior to the interview, participants were briefed that they would be asked to share their thoughts and opinions on the statements that were unclear or difficult to comprehend [25]. Participants were encouraged to engage with the researcher by reacting to pieces of the document and explain if they found the items and/or content confusing or unfamiliar.

### Data analysis

Collected data was audio-recorded and transcribed verbatim and was imported into QSR NVivo version 12 for data management and analysis [28]. In the last five interviews conducted, no new codes or themes were generated and thus saturation of data was achieved. The CI data were analysed in a multistep process. The initial analysis comprised open coding, with the intention of establishing codes based on participant feedback and suggestions. Thereafter, a second analysis involved axial coding to identify patterns in the codes for each item based on participant feedback and to categorise it under broader themes. A template thematic analysis technique was utilised to extract themes from the date collected via the CTA procedure. Template thematic analysis uses a priori code frames to analyse and report on the data. A priori codes are usually taken from interview questions as a way of initially organising the data. Template analysis commonly uses the questions as a skeleton code frame for thematic analysis [29]. Items generated from all interviews (including the initial patient interviews) and the literature relating to coping with cancer were collated into the interview statements. Additional items were also generated based on emerging themes identified during the CI data analysis. The identified themes were then refined and operationalised into statements corresponding to each theme.

### Investigative team review

During multiple sittings, the supervisory team from clinical and research backgrounds, reviewed the modified statements to establish a final set of items. A qualitative researcher who had collaborated closely with community groups, a health researcher with research expertise in qualitative study designs who had expertise in developing quality of life measures, a health researcher whose expertise lies in the areas of health service redesign and translation, a gynaecologist, and a higher degree research student. Team meetings formed a fundamental part of the questionnaire development phase. In compliance with all collective feedback and suggestions, supervisory team meetings were held to determine necessary changes and achieve a consensus on the modifications.

## Results

### Participants

Fourteen participants took part in individual telephone or video interviews with a mean duration of one hour and fifteen minutes (range of 30 min to one hour 50 min). Interviews for one participant were completed over two occasions for their convenience, and the initial interview which provided pilot data was also incorporated into the dataset. Feedback collected from the pilot interview informed modification of the statements’ response scale to be used in the subsequent interviews. Participants varied in age, employment and marital status but all lived in a metropolitan setting. Seven participants had been diagnosed over five years previously and two participants had received their diagnosis within a year of the interview. Among the 14 interviewees, five participants were undergoing active treatment because of recurrence of their cancer (Table 2).

**Table 2:** Description of study participants (n = 14)

Characteristics	No: of participants
**Age group (years)**	
50 to 59	5
60 to 69	5
70 to 79	4
**Current employment status**	
Currently looking for work	1
Employed (Casual, Part-time, Full-time, Self-employed)	5
Home duties	1
Retired	7
**Education**	
High school	5
TAFE certificate	4
University degree (undergraduate/postgraduate)	5
**Employment status before diagnosis**	
Employed (Casual, Part-time, Full-time, Self-employed)	10
Home duties	2
Retired	2
**Marital status**	
Married or de facto	8
Separate or Divorced	4
Single/Never married	1
Widowed	1
**Treatment status**	
Not on treatment	9
Currently on Treatment	5
**Length of time since diagnosis**	
6 months–1 year	2
2–4 years	5
≥ 5 years	7
**Cancer recurrence (yes)**	5

*Kindly include Table 2 in the text file (pg. 9) during production

### Draft questionnaire examined using CI and CTA

Draft items were classified into the domains identified in our previous qualitative study. Diagnosis and Treatment-related (chemotherapy, surgery and complementary therapies), relationships with family/friends, financial aspects, health services and interactions with health professionals, and coping strategies. Challenges related to diagnosis and treatments were documented. Key themes such as physical wellbeing, emotional wellbeing, relationships with family/friends, health services and interactions with health professionals, and coping strategies were identified as related to living with an OC diagnosis across the clinical journey. A 5-point Likert response scale was created for the items.

The draft questionnaire acknowledged all data from the original qualitative study and included a set of questions to collect participant demographic and cancer history information. The next four sections focused on challenges related to diagnosis and treatment and included skip questions which directed participants to appropriate sections based on the responses given, with the response scale measuring the severity of challenges. The remaining sections with items related to HRQOL, satisfaction with services and coping strategies were provided with a response scale of frequency (Table 3).

**Table 3:** Total number of items per questionnaire section prior to modification based on CI data

	Items (*n*)
Demographics	10
Cancer History	5
Section 1: Clinical Diagnosis	4
Section 2: Chemotherapy	5
Section 3: Surgery	5
Section 4: Complementary Therapies	2
Section 5: Emotional Wellbeing	52
Section 6: Financial Wellbeing	10
Section 7: Health Services	15
Section 8: Communicational & Informational Challenges	13
Section 9:	
Seeking help & Coping Strategies	10
Resilience	15

### Refinement of HRQOL items

A multistep analysis process was then used to classify how the items could be modified, using four broad themes. Refinement of HRQOL items is illustrated with examples below.

### Item intent and clarity

Responses for several items were inconsistent with the item intent, failing to interpret the researcher’s objectives. Specifically, for some items, participants reported, “I do not understand what this means” or “I couldn’t get to the meaning of the question”. For example, one original item was “I felt frustrated during and/or after receiving a treatment”. A majority of the participants comprehended “frustrated” in terms of the treatment received, but the researcher’s intent was to measure “frustrated” in relation to a participant’s activities whilst undergoing treatment. With participant input, the item was modified to “I felt frustrated that I could not take part in usual activities during and/or after treatments”.

### Item wording

Difficulties with comprehension of the meaning of some items was identified. For instance, the statement “I feel valued because I can still contribute to the workforce” created confusion in the participant. One particular response was “I am not working, but I still feel valued. I would put not applicable for that one. But I would put strongly disagree because I feel valued because of work”. To better structure it, the item was modified to “I have felt valued because of the work that I can do (home, workforce)”.

Other items were considered vague by participants and difficulties arose in communicating the researcher’s intent of the items to the participants. For example, the item “My family/friends have reacted unexpectedly to my illness” could have been interpreted as a positive or negative experience. With participant feedback, the item was modified to “My family/friends have reacted unexpectedly (in a negative way) to my illness”.

### Item relevance and context

Some items had little relevance to the participants’ age. These items included: “I have been embarrassed by the way my body has changed” and “I have felt less feminine because of my illness”. Moreover, several items specific to emotional wellbeing were not relevant to participants who were under surveillance and not receiving treatment. For example, when asked about “I have felt sick and unwell due to the side-effects of treatments I have experienced”, participants replied saying “that’s hard because during the chemo, I felt unwell and sick but since the chemo, I have had no problem”.

Finally, the complexities of the wording some items made it difficult for participants to respond precisely. For example, one original item was “My family/friends are generally supportive of me at this time”. However, based on the participant’s perspective, the support received from family and friends could have been different. Thus, the item was separated into two items. In particular, one participant couldn’t comprehend the context of the item within the social domain. Initially, the statement was developed as “I have felt isolated socially because of my illness. The participant could not, however, answer in what context the word "socially" meant. The item was subsequently changed to “I have found it difficult to connect socially with people because of my illness (e.g. at work, in public)”.

### Refinement of items describing contextual factors for HRQOL

The qualitative dataset contained additional themes of disease/treatment and financial issues, communication with health professionals and coping strategies. These themes were developed into three sets of questions that reflected contextual factors for HRQOL: patient symptoms, satisfaction with health services and strategies for self-sufficiency and resilience. These items were refined using the same multistep analysis process and examples are presented below.

### Item wording

Some items related to technical terms that were complex to understand. For example, concerns were raised on specific terminologies such as “cancer recurrence”, “mucositis”, “full cycle of chemotherapy”, “complementary therapies”. In response, clear definitions were constructed (Additional file 3).

### Item relevance and context

Relevance of specific items was age dependent. For instance, questions regarding “sudden onset of menopause” and “inability to have children” had no impact on the wellbeing of many participants because they had already undergone menopause prior to receiving their OC diagnosis. Thus, a “not applicable” response column was included to the patient symptom section. Since there were items of little relevance across the questionnaire and upon joint agreement amongst the supervisory team, a timeline was provided for every section.

The context of the item “There is a lack of financial assistance with practical support” was unclear as one participant expressed, “I haven’t had to seek that out. So, I don’t really know. I haven’t needed it but to listening other ladies I think there probably is a need for it”. However, the researcher’s intent was to know whether the participants had any challenges accessing the services or not. The item was modified as “There has been a lack of practical support offered to me”. In addition, the item “I maintained a sense of gratitude” seemed out of context to a participant. The item was modified to “I have maintained a sense of gratitude for what I am able to do/achieve”.

### Questionnaire construct

Some participants mentioned that the response format type for HRQOL and wellbeing items could be improved as responses could have reflected upon frequency, rather than merely agreeing/disagreeing to a statement. For instance, participants responded by using expressions such as “at times” or “sometimes” and found it difficult to merely agree/disagree to the items. Thus, a frequency format of Always/Often/Sometimes/Rarely/Never was applied to sections that measured aspects of HRQOL and wellbeing.

In summary, the CI and CTA processes informed substantial item reconstruction to achieve acceptable content validity for this participant group. The questionnaire in its entirety was restructured: including 67 items which were modified, 10 items were condensed and merged into appropriate sections, 66 items were deleted, and 15 items were added. The items describing HRQOL were grouped, forming an HRQOL instrument named the OVArian cancer health related Quality Of Life (OVAQOL) scale. This scale comprises items that contributed conceptually to three HRQOL domains: physical wellbeing (n = 7), emotional wellbeing (n = 21) and social wellbeing (n = 10). The rich original qualitative dataset and the comprehensive interview and analysis processes informed the development of accompanying sets of questions on demographic characteristics, disease and treatment status, patient satisfaction with healthcare services and patient resilience (Table 4).

**Table 4:** Final structure and number of items in the questionnaire with examples of OVAQOL items

	Items (*n*)
**Section 1: Demographics**	14
**Section 2: Disease and Treatment related symptoms**	26
**Section 3: OVArian cancer health related Quality Of Life (OVAQOL) scale**	7
**Physical Wellbeing**	
*Example:-*	
“felt sick due to treatment side-effects”	
“bothered by the symptoms”	
“frustrated by not being able to exercise”	
“difficult to care for my family and/or friends”	21
**Emotional Wellbeing**	
*Example:-*	
“afraid of cancer coming back”	
“felt valued because of the work that I can do”	
“felt less self-worth”	
“worried about loss of income”	10
**Social Wellbeing**	
*Example:-*	
“difficult to understand carer’s/partner’s feelings”	
“family has been generally supportive”	
“found it difficult to connect socially with people”	
“partner needed more self-time”	
**Section 4: Satisfaction with Health Services**	11
**Section 5: Resilience**	22

*Kindly include Table 4 in the text file (pg.15) during production

## Discussion

Using a CI approach, the purpose of this study was to refine and validate the wording of the items extracted from the data collected during a previous qualitative study with 14 women diagnosed with ovarian cancer. The draft items were administered through discussion, along with questions about the meaning and wording of the items. All items were adjusted based on participant input. The final set of items comprised 7 physical wellbeing items, 21 emotional wellbeing items and 10 social wellbeing items were identified as having meaning to the participants. They reflected a range of personal experiences, creating a new tool specific to women diagnosed with ovarian cancer. The CI process established content validity for the tool and highlighted the fundamental role of CI during questionnaire development. Feedback and suggestions provided by the participants. Following modification and evaluation, evidence for the content validity of the items was generated. Going forward, this new questionnaire has capacity to measure outcomes in women with OC and contribute to improving their health outcomes across the survivorship trajectory [30], and is ready for further validation.

Originally, the draft instrument consisted of 52 HRQOL items, and thereafter, has been reduced to 38 items. Since the questionnaire seeks to measure disease and treatment specific HRQOL outcomes of women diagnosed with OC, the items and its contents should reflect the purpose of measuring HRQOL. HRQOL is a multidimensional construct that measures the impact of a disease on physical, psychological and social relations aspects on a person’s life [31] as defined by the World Health Organisation [32]. Upon modification and evaluation, the items were examined and categorised into physical/functional, emotional and social domains consistent with this definition. The concept of HRQOL does not measure aspects such as job or income security [33]. Thus, items pertaining to accessing information in relation to financial needs were included in the patient satisfaction section with health services, while an item that measures psychological distress in relation to loss of income was merged with HRQOL emotional domain.

Our study findings illustrate how disease/treatment related symptoms had substantial impacts on the HRQOL of our study population, consistent with previous studies [34, 35]. As a consequence, these symptoms in turn affect the ability and capability to perform tasks either it be usual activities or professional [36]. Emergence of physical wellbeing constituted one of the important HRQOL domain. Included statements pertained to both physical health and functioning status of patients diagnosed with OC. The statements illustrated the impacts of symptoms from the patient perspective. Items related to “fatigue” and “difficulty sleeping” measure patient health while “participation in usual activities” focuses to measure patient functional ability. Similar to our findings, other studies have also revealed that people with chronic illness struggle with daily life tasks by being dependent on others [37, 38], revealing that it is important to measure physical domain of HRQOL for patients with OC.

Emotional wellbeing is a fundamental component of HRQOL instruments. Items including “depression”, “anxiety”, “fear of recurrence” were included in OVAQOL. Inclusion of such items enable evaluation of psychological distress experienced by women whether it be disease and/or treatment related by how severely it has impacted their HRQOL. By measuring psychological and emotional well-being and if difficulties are identified, clinicians could refer their patients to appropriate services and supports including counselling. Responses to PROMs questions help hospitals and healthcare services provide the care that patients need and want. These measures aim to fill a vital gap in our knowledge about outcomes that matter to patients [39]. In addition, it enables the researcher to identify whether such items impact other domains of HRQOL as previous research shows that psychological distress is related to poor performance status [40].

Personalized medicine should also focus on a patients’ cognitive, psychological, familial, and societal factors that influence clinical decisions. We believe that assessing the psycho-emotional status of OC patients and evaluating their resilience using OVAQOL will assist healthcare providers in conveying information in a tailored manner based on patients' characteristics, thereby providing suggestions and alerts; and potentially increase patient participation in the consultation process, as well as their satisfaction and involvement in their treatment decision-making processes.

Social functioning is also an essential component of HRQOL, particularly in relation to support provided by the family and friends which is emphasized in other similar qualitative studies [41]. In turn, social relations and mental wellbeing domains are interconnected. In a 2001 study of individuals with breast cancer, Kornblith and colleagues identified that women with low levels of support either it be through family, friends or professional, had higher levels of psychological distress throughout their clinical journey [42].

The initial qualitative dataset included important information describing factors related to the women’s HRQOL, including their satisfaction with health services, help-seeking throughout their clinical journey and resilience. In a recent study, it was indicated that HRQOL for those affected with systemic lupus erythematosus found a positive association between patient health care satisfaction and health status, possibly due to supports in the physical, emotional and social domains [33]. Recent studies also show that there is a direct relationship between resilience, life satisfaction and wellbeing on those living with chronic illness [43–45] and indicates the importance of measuring the impact of such variables on wellbeing and HRQOL. Thus, based on our findings and supporting information from various literature, relevant items associated with health services and informational challenges were collected into a module of questions describing ‘satisfaction with health care services’. Items that measured various coping strategies and resilience were collated into a ‘resilience’ module of questions to enable measurement of the self-empowerment and self-sufficiency strategies used by women during these difficult times.

Based on the development processes, the researchers believe that the content of OVAQOL truly reflected the consumer voice as was captured through the preceding qualitative study and the current CI procedures, each with patient involvement. This had not been performed to this extent in the development of existing OC HRQOL instruments suggesting limited patient involvement during the developmental stages of these PROMs. Therefore, the number of items in each HRQOL domain is larger compared to QLQ-OV28 and FACT-O. For example, EORTC QLQ-C30 has only four items and FACIT FACT-G has six items in the emotional domain, while OVAQOL measures 21 different aspects of emotional well-being, all of which were obtained by the individual experiences of women diagnosed with OC. In addition, there is some uncertainty regarding their factor structures. For example, low correlations are observed in several studies suggest that the social subscales of the EORTC-QLQ-C30 and FACIT FACT-G do not measure the same construct [46].

Consumer involvement in PROM development is essential as it is most appropriately women who have experienced OC who can determine outcome relevance and comprehensibility of the instrument [47, 48]. Previous studies have indicated that lack of patient involvement affects the sensitivity, validity and response of the questionnaire tool [49, 50], in which a 2017 study showed patient involvement had not increased in PROM development over time [19].

### Limitations

The study methodology itself is one of the main strengths of this study. Utilization of CI not only aided in the refinement of the statements in the tool, but also enabled identification of limitations which in turn assisted in the modification of the questionnaire construct that defines HRQOL. This study also had certain limitations. The onset of COVID-19 during the course of this study hindered the participant recruitment process, thereby impacting the study progress. Administering CI to a small sample size contributed to the second limitation as sampling variation could have increased with even more participant recruitment where evaluating and reviewing the contents of the items could have improved. Even though research has suggested that it is ideal to recruit between seven to 10 participants to check and confirm participant’s item comprehension, the variability in the participant number depends upon factors such as maximum sampling variation, questionnaire complexity and participant understanding of the items [51].

## Conclusion

The current study utilized a systematic process to develop an OC specific PROM and highlights the value of CI for questionnaire item modification and content validity. This tool has potential to monitor patients' psycho-emotional condition in relation to their quality of life with greater granularity which could help healthcare practitioners more effectively identify and manage patients’ symptoms and concerns and track their progress over time. Validation of the PROM in a larger sample and evaluation of its psychometric properties is an essential next research project.

## Supplementary Information

**Additional file 1**: Published work of preceding research. Prior to this study, a qualitative study with women with OC was conducted, which identified key experiences and priorities such as challenges related to diagnosis and treatment, adjustments in their relationships with family and/or friends, and comments on useful coping strategies. These findings were expanded into a set of items that could be useful in developing a values-based OC PROM.

**Additional file 2**: Cognitive Interviewing Questionnaire. The questionnaire was created specifically for this study. CI using the CTA procedure was used to test identified statements from our previous qualitative study. Set of draft items was administered via telephone, Zoom and WhatsApp app on fourteen women with OC.

**Additional file 3**: Consolidated criteria for reporting qualitative studies (COREQ): 32-item checklist. The study was carried out in accordance with the COREQ 32-item checklist, which assisted researchers in reporting important elements of the qualitative research, study design, study context, findings, evaluation, and interpretations.

## Data Availability

The datasets used and/or analysed during the current study are currently safely stored in NVivo software within researcher’s work laptop and are available from the corresponding author on reasonable request.

## Abbreviations

CCWA: Cancer Council Western Australia
CI: Cognitive Interviewing
CTA: Concurrent Think-Aloud
EORTC QLQ-C30: European Organization for Research and Treatment of Cancer Quality of Life Questionnaire for Cancer Patients
EORTC QLQ-OV28: European Organization for Research and Treatment of Cancer Quality of Life Questionnaire-Ovarian
FACIT FACT-G: Functional Assessment of Chronic Illness Therapy Functional Assessment of Cancer Therapy–General
FACT-O: Functional Assessment of Cancer Therapy–Ovarian
FOSI: Functional Assessment of Cancer Therapy–ovarian cancer symptom index
HRQOL: Health-Related Quality of Life
MOST: Measure of Ovarian Symptoms and Treatment concerns
OC: Ovarian Cancer
OCA: Ovarian Cancer Australia
OVAQOL: OVArian cancer health related Quality Of Life scale
PROMs: Patient Reported Outcome Measures
UNDA: University of Notre Dame Australia
