# Supplementary material for: Employing cognitive interviewing to evaluate, improve and validate items for measuring the health-related quality of life of women diagnosed with ovarian cancer
Source: BMC Womens Health. 2022 Sep 27;22:391. doi: 10.1186/s12905-022-01966-w (PMC9512969; doi:10.1186/s12905-022-01966-w)
Supplement: Supplementary file 2 — Additional file 2: Cognitive Interviewing Questionnaire. The questionnaire was created specifically for this study. CI using the CTA procedure was used to test identified statements from our previous qualitative study. Set of draft items was administered via telephone, Zoom and WhatsApp app on fourteen women with OC. [file 12905_2022_1966_MOESM2_ESM.docx]

INTRODUCTORY QUESTIONS

**Demographics**

Q1. What is your name? ____________________________

Q2. What is your current age (years)? ____________________________

Q3. What is your suburb/postcode? ____________________________

Q4. What is the highest level of education you have COMPLETED?

| Some primary school |  | Completed primary |  | Some high school |  |
| --- | --- | --- | --- | --- | --- |
|  |  |  |  |  |  |
| Completed high school |  | TAFE certificate |  | University undergraduate degree |  |
|  |  |  |  |  |  |
| University postgraduate degree |  | Other (specify) ____________________________ | | | |

Q5. What is your MARITAL STATUS?

| Single / never married |  | Married / de facto |  | Separated / divorced |  |
| --- | --- | --- | --- | --- | --- |
|  |  |  |  |  |  |
| Widowed |  |  |  |  |  |
|  |  |  |  |  |  |

Q6. Do you have children? Yes No

Q6a. If yes, please specify their gender and age

| **Child** | **Gender** | **Age** |
| --- | --- | --- |
| Child 1 |  |  |
| Child 2 |  |  |
| Child 3 |  |  |
| Child 4 |  |  |
| Child 5 |  |  |
| Other children |  |  |

Q7. Which of the following best describes your employment status at time of your diagnosis?

| Full time paid work |  | Part time paid work |  | Home duties |  |
| --- | --- | --- | --- | --- | --- |
|  |  |  |  |  |  |
| Studying |  | Currently looking for work |  | Retired |  |
|  |  |  |  |  |  |
| Disability pension |  | Other (specify) ____________________________ | | | |

Q7a. Which of the following best describes your current employment status?

| Full time paid work |  | Part time paid work |  | Home duties |  |
| --- | --- | --- | --- | --- | --- |
|  |  |  |  |  |  |
| Studying |  | Currently looking for work |  | Retired |  |
|  |  |  |  |  |  |
| Disability pension |  | Other (specify) ____________________________ | | | |

Q8. Do you have a health care card? **(please circle one)** Yes / No/ Not sure

**Cancer History**

Q9. Were you previously diagnosed with other cancer types before Ovarian Cancer (e.g. breast, colorectal)?

**(please circle one)** Yes / No

If Yes, could you please specify? (Type of cancer(s) & Time when you received a diagnosis (months/years)) ______________________________________________________________________________________________________________________________________________________________________________________________________

Q10. Were you aware of any genetic or hereditary issues that you might have had prior to receiving Ovarian Cancer Diagnosis (e.g. BRCA gene; Breast/Colorectal/Ovarian cancer in family members , etc)?

**(please circle one)** Yes / No

If Yes, please provide the details.

______________________________________________________________________________________________________________________________________________________________________________________________________

Q11. When did you receive your diagnosis of Ovarian Cancer (month/year)? ___________

Q12. At what stage was the cancer when you received the diagnosis?___________

Q13. Have you had a cancer **recurrence**? If yes, have you received any treatments? _______________________

**Section 1: Clinical Diagnosis**

Q1.

|  | **Yes** | **No** | **Don’t know** | **Can’t remember** |
| --- | --- | --- | --- | --- |
| My ovarian cancer was diagnosed accidentally (e.g. during surgery, emergency presentation at the Emergency Department at the hospital) |  |  |  |  |

Q2.

|  | **Yes** | **No** | **Don’t know** | **Can’t remember** |
| --- | --- | --- | --- | --- |
| Had no obvious signs and symptoms |  |  |  |  |

**If the answer to Q2 is Yes, please go to Section 2, page.6.**

Q3. The following questions are about the disease signs and symptoms and symptom presentation **challenges you may have experienced prior to receiving a diagnosis.** Please mark one answer per statement.

For each statement, please rate the overall impact of following symptoms on your well-being. *Before I received my diagnosis, I…..*

|  | **Severe** | **Moderate** | **Mild** | **None** |
| --- | --- | --- | --- | --- |
| Had abdominal and bowel pain/discomfort (e.g. bloating, constipation) |  |  |  |  |
| Had joint pain |  |  |  |  |
| Had menopausal symptoms |  |  |  |  |
| Had pain in my vaginal area |  |  |  |  |
| Had the sensation of needing to go to the toilet more frequently |  |  |  |  |
| Experienced eye issues (eg. Itchy eyes) |  |  |  |  |
| Felt fatigued (e.g. tiredness, lack of energy) |  |  |  |  |
| Gained weight |  |  |  |  |
| Wasn’t really aware of my symptoms as part of ovarian cancer (i.e. I thought they were due to something else, e.g. menopausal symptoms) |  |  |  |  |

Q4. Please describe **other challenges** that you might be experiencing or have experienced in terms of your physical health:

|  | **Severe** | **Moderate** | **Mild** | **None** |
| --- | --- | --- | --- | --- |
| **A-** |  |  |  |  |
| **B-** |  |  |  |  |
| **C-** |  |  |  |  |
| **D-** |  |  |  |  |

**Section 2: Chemotherapy**

Q1. Have you received chemotherapy in the past five years? (please circle one) Yes/No

**If the answer is No, please go to Section 3, page.8.**

Q2. How many full cycles of chemotherapy treatment have you received to date? _______________________

Q3. When was the last cycle of chemotherapy you have completed (month/year)? _______________________

Q4. The following questions specifically ask about the ***challenges*** (e.g. side effects, chemotherapy administration) you have experienced **during or after receiving** **chemotherapy.** Please mark one answer per statement.

For each statement, please rate the overall impact of following side effects on your well-being. *During my chemotherapy, I experienced…..*

|  | **Severe** | **Moderate** | **Mild** | **None** |
| --- | --- | --- | --- | --- |
| Back pain (mild, moderate, severe) |  |  |  |  |
| Bloating with Cisplatin |  |  |  |  |
| Blood clots due to collapsed veins |  |  |  |  |
| Bowel problems (e.g. diarrhoea, constipation, bloating) |  |  |  |  |
| Carboplatin side effects |  |  |  |  |
| Cardiac arrest at time of chemo administration |  |  |  |  |
| Collapsed veins |  |  |  |  |
| Fatigue (tiredness, lack of sleep) |  |  |  |  |
| Frequent urgency to pass urine |  |  |  |  |
| Hair loss |  |  |  |  |
| Issues receiving chemo as an out-patient (e.g. chemo at home) |  |  |  |  |
| Issues receiving PICC line treatment |  |  |  |  |
| Joint pain |  |  |  |  |
| Loss of appetite |  |  |  |  |
| Medication related side effects (e.g PEG allergy, Phenergan) |  |  |  |  |
| Memory loss |  |  |  |  |
| Mouth ulcer/s |  |  |  |  |
| Mucositis |  |  |  |  |
| Nausea |  |  |  |  |
| Neuropathy |  |  |  |  |
| Pain |  |  |  |  |
| Poor balance |  |  |  |  |
| Taste disorder with Olaparib |  |  |  |  |
| Tinnitus |  |  |  |  |

Q5. Please describe **other** chemotherapy related challenges you have experienced: ­­­­­­­­­­­

|  | **Severe** | **Moderate** | **Mild** | **None** |
| --- | --- | --- | --- | --- |
| **A-** |  |  |  |  |
| **B-** |  |  |  |  |
| **C-** |  |  |  |  |
| **D-** |  |  |  |  |

**Section 3: Surgery**

Q1. Have you undergone a treatment related surgery in the past five years? (please circle one) Yes/No

**If the answer is No, please go to Section 4, page.9.**

Q2. How many surgeries have you received to date? _______________________

Q3. When was the last surgery you have undergone (month/year)? _______________________

Q4. We would like to know any concerns and/or challenges that you may have faced after undergoing **surgery.** Please mark one answer per statement.

For each statement, please rate the overall impact of the following challenges on your well-being*. I experienced…..*

|  | **Severe** | **Moderate** | **Mild** | **None** |
| --- | --- | --- | --- | --- |
| Temporarily cessation of chemotherapy due to surgery |  |  |  |  |
| Anaesthetic issues during the surgery (e.g. fault of Anesthesiologist) |  |  |  |  |
| Fluid filled stomach post-surgery |  |  |  |  |
| Weight loss |  |  |  |  |
| Sudden onset of menopause |  |  |  |  |
| Difficulties with Stoma Bag |  |  |  |  |
| Inability to have children |  |  |  |  |

Q5. Please describe **other** surgery related challenges you have experienced: ­­­­­­­­­­­

|  | **Severe** | **Moderate** | **Mild** | **None** |
| --- | --- | --- | --- | --- |
| **A-** |  |  |  |  |
| **B-** |  |  |  |  |
| **C-** |  |  |  |  |
| **D-** |  |  |  |  |

**Section 4: Complementary therapies**

Q1. Have you received any alternative treatments? (please circle one) Yes/No

**If the answer is No, please go to Section 5, page.10.**

Q2. Please provide the details of any alternative cancer treatments you have received in the past five years:-

_______________________________________________________________________________________________________________________________________________________________________________________________________________________________________________________________________________________________________________________________________________________________________________________________________________________________________________________________________________________________________________________________________________________________________________________

**Please complete all the following sections, from pages.10-16.**

**Section 5: Emotional Well-Being**

Now, we would like to ask you about your ***well-being in terms of your feelings/emotions.*** When we talk about emotional well-being we are talking about any feelings, worries or concerns that you may have had across the course of the disease.

Please tick one answer per statement. *Since my diagnosis of ovarian cancer….*

|  | **SA** | **A** | **D** | **SD** |
| --- | --- | --- | --- | --- |
| I am frustrated by not being active or able to exercise (as I used to) |  |  |  |  |
| I feel powerless at not making decisions myself (e.g. both small decisions and larger decisions) about my care |  |  |  |  |
| I feel that I have lost my sense of who I am |  |  |  |  |
| I feel that there is a lack of awareness around this disease by others |  |  |  |  |
| I feel there is little awareness of ovarian cancer outside of my family and close friends (e.g. lack of social awareness) |  |  |  |  |
| I feel valued because I can still contribute to the workforce (I am in paid employment) |  |  |  |  |
| I find it difficult to stay strong in front of others |  |  |  |  |
| I find it hard to stay strong for myself |  |  |  |  |
| I have been embarrassed by the way my body looks (e.g. having a colostomy bag, stoma bag) |  |  |  |  |
| I have been unwilling to accept my diagnosis |  |  |  |  |
| I have been worried about loss of income due to my illness |  |  |  |  |
| I have difficulty accepting the reality of my diagnosis |  |  |  |  |
| I have felt less feminine because of my illness |  |  |  |  |
| I have felt there was a lack of treatment choices offered to me |  |  |  |  |
| I have felt uncertainty when looking forward because of my illness |  |  |  |  |
| I have had concerns with medications that can have severe side effects |  |  |  |  |
| I have had difficulty in looking forward to the future |  |  |  |  |
| I have had emotional issues due to sudden menopause |  |  |  |  |
| I have had issues with not being able to have children (due to surgery) |  |  |  |  |
| I have lower self-esteem and feelings of self-worth due to my illness |  |  |  |  |
| There are some taboo topics (e.g. Euthanasia) that I have no one to talk to about |  |  |  |  |
| **CHALLENGING EMOTIONS** |  |  |  |  |
| I have been in shock about my diagnosis and illness |  |  |  |  |
| I have felt angry about my illness |  |  |  |  |
| I have felt frustrated about my illness |  |  |  |  |
| I have felt stressed as a parent because of my illness |  |  |  |  |
| I have had negative feelings and emotions (e.g. stress, anger) when participating in Ovarian Cancer support groups because of my illness |  |  |  |  |
| I felt frustrated during and/or after receiving a treatment (e.g. not able to take part in activities that I used to do) |  |  |  |  |
| I have felt sick and unwell due to the side-effects of treatments I have experienced |  |  |  |  |
| I have felt stressed during and/or after receiving a treatment |  |  |  |  |
| **ANXIETY** |  |  |  |  |
| I have been anxious because of my illness |  |  |  |  |
| **DEPRESSION** |  |  |  |  |
| I have been diagnosed with depression in relation to my illness |  |  |  |  |
| I have felt downhearted and sad because of my illness |  |  |  |  |
| **FEAR** |  |  |  |  |
| I have felt afraid (of illness, treatment and prognosis) |  |  |  |  |
| I have felt afraid of dying from my illness |  |  |  |  |
| I have felt afraid that the cancer will come back again |  |  |  |  |
| **ISOLATION** |  |  |  |  |
| I felt like I was being categorised (e.g. physical appearance-hair loss) because of my illness |  |  |  |  |
| I have felt isolated socially because of my illness |  |  |  |  |
| **RELATIONSHIPS** |  |  |  |  |
| I found it difficult to understand my carer’s/partner’s feelings since my diagnosis |  |  |  |  |
| My family/friends are generally not supportive of me at this time |  |  |  |  |
| My family/friends have reacted unexpectedly to my illness |  |  |  |  |
| My partner and I experience a lack of intimacy since my diagnosis |  |  |  |  |
| My partner needs time for him/herself since my diagnosis |  |  |  |  |
| People that I was close to have withdrawn from me since my diagnosis |  |  |  |  |
| **HEALTH SERVICES** |  |  |  |  |
| I feel that I have been given false hope by health professionals |  |  |  |  |
| I feel that the health professionals are not listening to me (patient-centered care) |  |  |  |  |
| I feel that the health professionals have little empathy |  |  |  |  |
| I felt that health professionals lack treatment experience (ovarian specific) |  |  |  |  |
| I felt there is a lack of health professionals available |  |  |  |  |
| I have experienced unprofessional behaviour by health professionals |  |  |  |  |
| I have felt let down by the health system |  |  |  |  |
| I have felt that the health professionals are not really taking my needs into consideration (e.g. denial of patient centered care) |  |  |  |  |
| I lack confidence in health professionals |  |  |  |  |

**Section 6: Financial Wellbeing**

Q1. The following statements are about other ***practical challenges*** you may have faced during/after a particular treatment. We would like to know any financial challenges you may have faced that have impacted your well-being.

Please tick one answer per statement. *I experienced….*

|  | **SA** | **A** | **D** | **SD** |
| --- | --- | --- | --- | --- |
| Financial barriers due to living in a rural or remote place (e.g. travelling difficulties to receive a treatment) |  |  |  |  |
| Barriers to undergoing surgery due to work commitments |  |  |  |  |
| Difficulties with ambulance cover costs (insurance) |  |  |  |  |
| Difficulties with car parking whilst attending appointments at the hospital |  |  |  |  |
| Financial instability since my diagnosis |  |  |  |  |
| Lack of information regarding financial support (e.g. HBF, PBS) |  |  |  |  |
| Limited travel options for work or holiday (e.g. interstate, within the state) due to my treatment |  |  |  |  |
| Out of pocket expenses due to my medical costs (e.g. surgery, insurance cover issues, PET scan test, MRI tests) |  |  |  |  |
| A need for financial support (e.g. superannuation and/or disability pension support, private health insurance) |  |  |  |  |

Q2. Please describe **other** practical challenges (not mentioned above) that you have experienced: ­­­­­­­­­­­

|  | **SA** | **A** | **D** | **SD** |
| --- | --- | --- | --- | --- |
| **A-** |  |  |  |  |
| **B-** |  |  |  |  |
| **C-** |  |  |  |  |
| **D-** |  |  |  |  |

**Section 7: Health Services**

The following statements focus **on issues** you may have faced **in relation to the services** provided to you once you were diagnosed with ovarian cancer. **This includes any health services provided including those provided by your GP.**

Please tick one answer per statement and rate the overall impact of the following challenges on your well-being*.*

|  | **SA** | **A** | **D** | **SD** |
| --- | --- | --- | --- | --- |
| I feel there are less services provided to ovarian cancer patients compared to other higher profile cancer types (e.g. Breast, Prostate) |  |  |  |  |
| I have experienced poor medical decision making by the health professionals |  |  |  |  |
| I have had genetic testing (*BRACA* gene) |  |  |  |  |
| The health professionals I consulted about my symptoms did not consider ovarian cancer |  |  |  |  |
| There are inadequate support service programs (e.g. counselling) |  |  |  |  |
| There is a lack of financial assistance with practical support (e.g. house cleaning) |  |  |  |  |
| There is lack of communication between health professionals (e.g. GP and specialists) |  |  |  |  |
| There is lack of opportunity to participate in clinical trials for new treatments |  |  |  |  |
| There is lack of respite care for Ovarian Cancer patients (e.g. single parents) |  |  |  |  |
| **GP** |  |  |  |  |
| I feel that my GP was biased and did not take my concerns seriously because I am a woman |  |  |  |  |
| I felt that my GP contributed to the delay in my diagnosis |  |  |  |  |
| I had to be proactive and request that my GP refer me for testing |  |  |  |  |
| I presented at the hospital with specific symptoms (e.g. Abdominal discomfort, Fatigue, bloating) that had not been identified by my GP |  |  |  |  |
| My GP is actively involved throughout my diagnosis and illness |  |  |  |  |
| Overall, I feel that my GP is supportive of me and my illness |  |  |  |  |

**Section 8: Communication & Informational Challenges**

Now, we would like to know of any **communication challenges** you may have experienced in particular with health professionals (e.g. lack of patient centered care, poor medical decision making), including whether you have **had trouble getting information during active treatment.**

Please tick one answer per statement and rate the impact of following challenges on your well-being. *I have experienced communication challenges in….*

|  | **SA** | **A** | **D** | **SD** |
| --- | --- | --- | --- | --- |
| An ability to meet my language needs (e.g. English not the first language) |  |  |  |  |
| Receiving accurate information about my treatment and my illness in general (e.g. hospital staff, specialists, nurses) |  |  |  |  |
| Accessing central and accessible ovarian cancer specific information |  |  |  |  |
| Shared communication & information between health professionals |  |  |  |  |
| Shared communication & information between hospital departments |  |  |  |  |
| Shared communication & information with health professionals |  |  |  |  |
| Shared communication & information with the ambulance crew |  |  |  |  |
| Health professionals’ ovarian cancer knowledge |  |  |  |  |
| Health system information (e.g. lack of information provided after receiving a treatment, lack of follow-up after receiving treatment) |  |  |  |  |
| Inability to ask questions of those providing treatment and services |  |  |  |  |
| Getting information regarding the OC Resilience Kit |  |  |  |  |
| Treatment information (i.e. options and side effects of each) |  |  |  |  |
| Lack of willingness to listen to my concerns |  |  |  |  |

**Section 9: Seeking Help & Coping Strategies**

Now, we would like to know **what helped you to** **cope and improve your sense of wellness** once you (i) were diagnosed and/or (ii) received active treatment. This includes whether or not you sought help and what your main strengths were throughout your clinical pathway**.**

Please select one answer per statement and rate the impact of following challenges on your well-being. *I was able to cope better because …..*

|  | **SA** | **A** | **D** | **SD** |
| --- | --- | --- | --- | --- |
| I have prior medical knowledge and training (e.g. a health professional such as a doctor or nurse) |  |  |  |  |
| I adjusted my diet and lifestyle activities (e.g. physical activities, yoga, reflexology) |  |  |  |  |
| I aimed to stay positive throughout |  |  |  |  |
| I benefitted from available information (medical knowledge, information provided by HPs) |  |  |  |  |
| I drew strength from my spiritual beliefs (e.g. religion, connecting with nature) |  |  |  |  |
| I managed well with the support of my family and friends |  |  |  |  |
| I relied on being treated ‘normally’ by others |  |  |  |  |
| I sought informational help (e.g. Google, research, survivor stories) |  |  |  |  |
| I took symptom relief medications (e.g. Anti-Depressants) as complementary to my treatment |  |  |  |  |
| The information I received from organisations helped me manage my illness (e.g. Psychologist referrals, Cancer Council, Support groups) |  |  |  |  |

We would like to know how **resilient you believe that are/were during your treatment**.

Please mark one answer per statement and rate the impact of each statement on your well-being. *In regards to managing my illness…..*

|  | **SA** | **A** | **D** | **SD** |
| --- | --- | --- | --- | --- |
| I have had a determined mind |  |  |  |  |
| I benefitted from having medical knowledge (professional & training) |  |  |  |  |
| I drew strength from family and friends |  |  |  |  |
| I found time for myself |  |  |  |  |
| I gained adaptability to my illness |  |  |  |  |
| I had a positive attitude to the illness |  |  |  |  |
| I had a sense of humour |  |  |  |  |
| I kept calm |  |  |  |  |
| I looked forward to the future |  |  |  |  |
| I maintained a positive attitude in relation to my illness |  |  |  |  |
| I maintained a sense of gratitude |  |  |  |  |
| I maintained a sense of humour |  |  |  |  |
| I made plans to look forward |  |  |  |  |
| I refused to give up on myself |  |  |  |  |
| I took relief medication as an alternative (e.g. anti-depressants) |  |  |  |  |
