# Supplementary material for: Employing cognitive interviewing to evaluate, improve and validate items for measuring the health-related quality of life of women diagnosed with ovarian cancer
Source: BMC Womens Health. 2022 Sep 27;22:391. doi: 10.1186/s12905-022-01966-w (PMC9512969; doi:10.1186/s12905-022-01966-w)
Supplement: Supplementary file 3 — Additional file 3: Consolidated criteria for reporting qualitative studies (COREQ): 32-item checklist. The study was carried out in accordance with the COREQ 32-item checklist, which assisted researchers in reporting important elements of the qualitative research, study design, study context, findings, evaluation, and interpretations. [file 12905_2022_1966_MOESM3_ESM.docx]

*Consolidated criteria for reporting qualitative studies (COREQ): 32-item checklist.*

| **No** | **Item** | **Guide questions/description** | **Manuscript page numbers** |
| --- | --- | --- | --- |
| **Domain 1: Research team and reflexivity** |  |  |  |
| Personal Characteristics |  |  |  |
| 1. | Interviewer/facilitator | Which author/s conducted the interview or focus group? | “Recruitment procedures and study population”  Pg.6-7 |
| 2. | Credentials | What were the researcher's credentials? E.g. PhD, MD | Author details, Pg.1;  “Investigative team review”  Pg.9 |
| 3. | Occupation | What was their occupation at the time of the study? | Author details, Pg.1;  “Investigative team review”  Pg.9 |
| 4. | Gender | Was the researcher male or female? | Author details, Pg.1 |
| 5. | Experience and training | What experience or training did the researcher have? | Author details, Pg.1; “Recruitment procedures and study population”  Pg.6-7 |
| Relationship with participants |  |  |  |
| 6. | Relationship established | Was a relationship established prior to study commencement? | “Recruitment procedures and study population”  Pg.6-7 |
| 7. | Participant knowledge of the interviewer | What did the participants know about the researcher? e.g. personal goals, reasons for doing the research | “Recruitment procedures and study population”  Pg.6-7 |
| 8. | Interviewer characteristics | What characteristics were reported about the interviewer/facilitator? e.g. Bias, assumptions, reasons and interests in the research topic | “Recruitment procedures and study population”  Pg.6-7 |
| **Domain 2: study design** |  |  |  |
| Theoretical framework |  |  |  |
| 9. | Methodological orientation and Theory | What methodological orientation was stated to underpin the study? e.g. grounded theory, discourse analysis, ethnography, phenomenology, content analysis | “Methods”  Pg.6 |
| Participant selection |  |  |  |
| 10. | Sampling | How were participants selected? e.g. purposive, convenience, consecutive, snowball | “Methods”  Pg.6 “Recruitment procedures and study population”  Pg.6-7 |
| 11. | Method of approach | How were participants approached? e.g. face-to-face, telephone, mail, email | Data collection Pg.7-8 |
| 12. | Sample size | How many participants were in the study? | “Results-participants” Pg.9 |
| 13. | Non-participation | How many people refused to participate or dropped out? Reasons? | NA |
| Setting |  |  |  |
| 14. | Setting of data collection | Where was the data collected? e.g. home, clinic, workplace | Data collection Pg.7-8 |
| 15. | Presence of non-participants | Was anyone else present besides the participants and researchers? | “Recruitment procedures and study population”  Pg.6-7 |
| 16. | Description of sample | What are the important characteristics of the sample? e.g. demographic data, date | “Results-participants” Pg.9 |
| Data collection |  |  |  |
| 17. | Interview guide | Were questions, prompts, guides provided by the authors? Was it pilot tested? | “Methods”  Pg.6 “Recruitment procedures and study population”  Pg.6-7; Data collection Pg.7-8 |
| 18. | Repeat interviews | Were repeat interviews carried out? If yes, how many? | NA |
| 19. | Audio/visual recording | Did the research use audio or visual recording to collect the data? | Data collection Pg.7-8 |
| 20. | Field notes | Were field notes made during and/or after the interview or focus group? | “Methods”  Pg.6 “Recruitment procedures and study population”  Pg.6-7; Data collection Pg.7-8 |
| 21. | Duration | What was the duration of the interviews or focus group? | “Results-participants” Pg.9 |
| 22. | Data saturation | Was data saturation discussed? | Data analysis Pg.8 |
| 23. | Transcripts returned | Were transcripts returned to participants for comment and/or correction? | Data collection Pg.7-8; Data analysis Pg.8 |
| **Domain 3: analysis and findings** |  |  |  |
| Data analysis |  |  |  |
| 24. | Number of data coders | How many data coders coded the data? | “Draft questionnaire examined using CI and CTA Pg10-11 |
| 25. | Description of the coding tree | Did authors provide a description of the coding tree? | “Draft questionnaire examined using CI and CTA Pg10-11 |
| 26. | Derivation of themes | Were themes identified in advance or derived from the data? | Data analysis Pg.8 |
| 27. | Software | What software, if applicable, was used to manage the data? | Data analysis Pg.8 |
| 28. | Participant checking | Did participants provide feedback on the findings?  Reporting | Data collection Pg.7-8; Data analysis Pg.8; “Results-participants” Pg.9 |
| Reporting |  |  |  |
| 29. | Quotations presented | Were participant quotations presented to illustrate the themes / findings? Was each quotation identified? e.g. participant number | Pg.11-13 (‘results’ section), anonymous participant identity |
| 30. | Data and findings consistent | Was there consistency between the data presented and the findings? | Discussed in ‘results’ and ‘discussion’ section |
| 31. | Clarity of major themes | Were major themes clearly presented in the findings? | Discussed in ‘results’ and ‘discussion’ section |
| 32. | Clarity of minor themes | Is there a description of diverse cases or discussion of minor themes? | Discussed in ‘results’ and ‘discussion’ section |
